# eHealth Tools Supporting Early Childhood Education and Care Centers to Assess and Enhance Nutrition and Physical Activity Environments: Protocol for a Scoping Review

**DOI:** 10.2196/52252

**Published:** 2023-10-24

**Authors:** Joyce Hayek, Katharine Elliott, Makayla Vermette, Lynne MZ Lafave

**Affiliations:** 1 Department of Health and Physical Education Mount Royal University Calgary, AB Canada

**Keywords:** early childhood education and care, childcare, nutrition, physical activity, healthy eating environment, eHealth, digital technology, health technology, digital public health, eating, diet, dietary, exercise, child, children, childhood, pediatric, pediatrics, digital health, early childhood educators, ECEC, knowledge synthesis, review methods, review methodology, scoping, mobile phone

## Abstract

**Background:**

Many children today are growing up in environments that predispose them to develop noncommunicable diseases. While no single preventive solution exists, evidence supports interventions in childcare settings for establishing good nutrition and physical activity behaviors as a “critical window” that could reduce the risk of developing noncommunicable diseases later in life. Emerging eHealth tools have shown potential in promoting best practices for nutrition and physical activity environments in early childhood education and care (ECEC) settings.

**Objective:**

The primary objective of this review is to map the breadth of available evidence on eHealth tools currently available to assess and support best practices for nutrition, physical activity, or both in ECEC settings and to highlight potential research directions.

**Methods:**

This scoping review will be conducted in accordance with the *Joanna Briggs Institute Manual for Scoping Reviews* with adherence to the PRISMA-ScR (Preferred Reporting Items for Systematic Reviews and Meta-Analyses Extension for Scoping Reviews) checklist guidelines. Eligibility is based on the Population, Concept, and Context criteria as follows: (1) early childhood educators (population); (2) eHealth (digital) technology, such as websites, smartphone apps, email, and social media (concept); and (3) measurement and intervention tools to support best practices for nutrition, physical activity, or both in ECEC settings (context). The information sources for this review are the bibliographic databases PubMed, Scopus, CINAHL Plus, ERIC, and Embase in English and French with no date restrictions. Following this, a scan of gray literature will be undertaken. The electronic search strategy was developed in collaboration with two librarians. Two independent reviewers will screen the titles and abstracts of all relevant publications against inclusion criteria, followed by a full-text review using a data extraction tool developed by the reviewers. A synthesis of included papers will describe the publication, assessment, and intervention tool details. A summary of the findings will describe the types of eHealth assessment tools available, psychometric properties, eHealth intervention components, and theoretical frameworks used for development.

**Results:**

Preliminary searches of bibliographic databases to test and calibrate the search were carried out in May 2023. Study selection based on titles and abstracts was started in August 2023. The developed search strategy will guide our search for gray literature. The findings will be presented in visualized data map format, waffle chart, or tabular format accompanied by a narrative discussion. The scoping review is planned for completion in 2024.

**Conclusions:**

A structured review of the literature will provide a summary of the range and type of eHealth tools available for ECEC programs to assess and improve nutrition environments, physical activity environments, or both in order to identify gaps in the current evidence base and provide insights to guide future intervention research.

**Trial Registration:**

Open Science Framework XTRNZ; https://osf.io/xtrnz

**International Registered Report Identifier (IRRID):**

DERR1-10.2196/52252

## Introduction

### Background and Rationale

Noncommunicable diseases (NCDs) are on the rise and represent the leading cause of disability and death worldwide [1]. In Canada, approximately 60% of deaths are due to chronic illnesses [2]. The latest statistics show that 1 in 3 Canadian adults live with at least one chronic disease, placing a significant physical, emotional, and financial burden on patients in addition to significant societal costs [3,4]. NCDs are largely preventable and share at least one predisposing risk factor related to unhealthy lifestyles, including physical inactivity and poor nutrition [5]. Thus, one avenue of prevention or treatment could be achieved through lifestyle modification of approaches such as good nutrition and physical activity.

Many children are growing up in environments that may predispose them to developing NCDs later in life. Research suggests that many preschool-aged children consume higher energy diets and do not meet the recommended intake for vegetables and fruits and have low levels of physical activity [6-10]. These findings are troubling, as lifestyle behaviors that contribute to the development of chronic diseases, such as eating habits and physical activity habits, are formed in the early years and often track into adulthood [11-13]. While the significance of early child development on adult health and lifelong outcomes is well acknowledged [14], health-promoting behavior interventions focused on healthy eating and physical activity in early childhood settings are an important area for critical inquiry to clarify optimal preventative strategies for support [15].

Early childhood education and care (ECEC) programs have been recognized as ideal settings to promote healthy behaviors. This is because an increasing number of children spend time in care during these formative years, and this represents a significant component of a young child’s weekly waking hours [16-18]. Evidence from a review on children’s physical activity correlates illustrates that on average physical activity levels are significantly associated with the ECEC setting they attend [19]. The influence on the development of eating behaviors and dietary intake in young children is also strongly influenced by educators and the ECEC setting [20-22]. As a result, ECEC centers are in a unique position to encourage and facilitate healthy behaviors in young children and represent a rich opportunity for interventions aiming at fostering healthier habits in children [23]. Moreover, recent studies suggest that professional development for childcare providers on nutrition and physical activity best practices can positively influence health-related practices and outcomes in children [24].

The proliferation of the internet along with the rapid growth and availability of digital technologies has provided a new platform for health interventions. eHealth refers to the use of information technology, including the internet, digital gaming, virtual reality, and robotics, in the promotion, prevention, treatment, and maintenance of health [25]. eHealth tools include digital technologies such as the web, smartphone apps, SMS text messaging, and social media modalities that may be used for measurement or intervention within a health improvement paradigm [26]. Among studies aiming at fostering healthier habits, many have adopted the eHealth modalities, including interventions targeting ECEC eating and activity practices. eHealth tools are becoming more popular as they can deliver information in various forms and have the potential to reach large audiences at low cost [27,28].

A preliminary search of PROSPERO, PubMed, the Cochrane Database of Systematic Reviews, and *JBI Evidence Synthesis* was conducted, and no current or in-progress scoping reviews or systematic reviews in the area of eHealth tools in the ECEC setting were identified. Previous reviews have primarily focused on examining the effectiveness of preschool-focused nutrition and physical activity interventions delivered face-to-face [29] or eHealth interventions in children and adolescents but not in preschool-aged children [27]. A previous review looked into health behavior resources for childcare educators but limited the focus to the Canadian context and did not consider international evidence [30]. Additionally, the authors explored both print and web-based materials [30]. With the rapid development of digital interventions, Prowse and Carsley [31] emphasize the need to examine those interventions to establish best practices for eHealth nutrition interventions for younger children. Hence, the specific focus of our review is on eHealth technologies. Furthermore, previous reviews emphasize the role of settings in the successful implementation of health promotion interventions [32,33]. For instance, in their review evaluating the success of childhood obesity interventions by setting, Flynn et al [32] found that interventions aimed at improving the food environment delivered in the ECEC setting were effective in improving childhood outcomes. Another important construct to consider is the theoretical basis of interventions. In a recent review conducted by Kracht et al [27] examining eHealth and mobile health interventions in children and adolescents, the authors underscored the importance of reporting on the theoretical underpinnings of eHealth interventions. The use of a theoretical framework in behavior change interventions was associated with increased effectiveness and maintenance of the desired behavior [28,31,34]. Additionally, the use of theory was noted as enabling potential replication for future intervention development [35]. Consequently, this underpins the importance of reporting and identifying the theoretical basis used to develop a given eHealth tool.

### Rationale and Objectives

For these reasons and given that no reviews on the use of eHealth tools developed for educators in ECEC settings were found, we are undertaking a scoping review in this area. This scoping review will allow a broad examination of the literature in this emerging field and provide directions for future systematic reviews.

Our broad objective is to explore which eHealth tools are available to assess and support best practices for nutrition, physical activity, or both in ECEC environments and to highlight potential future research directions. To map the breadth of the available evidence, the scoping review will address the following specific objectives: (1) to identify the existing eHealth tools that are used to assess or improve nutrition environments, physical activity environments, or both in ECEC centers; (2) to describe the components of the eHealth tools, including technology type (eg, websites, smartphone apps, and social media) and health purposes (eg, nutrition evaluation and movement promotion); (3) to describe the psychometric evidence provided; (4) to report the theoretical foundations used in developing the eHealth tools; and (5) to identify any evidence gaps.

## Methods

### Study Design

This scoping review will be conducted in accordance with the Joanna Briggs Institute methodology for scoping reviews [36] and follows the PRISMA-ScR (Preferred Reporting Items for Systematic Reviews and Meta-Analyses Extension for Scoping Reviews) guidelines [37].

### Ethical Considerations

No ethics approvals are required since the planned study is an assessment of the literature and will not include human or animal participants.

### Protocol and Registration

This protocol was written before the study commenced (ie, before the electronic literature search was performed). The protocol for this scoping review is registered on Open Science Framework (XTRNZ) [38].

### Eligibility Criteria

#### Overview

The eligibility for our scoping review is based on the Population, Concept, and Context framework (Textbox 1).

**Eligibility criteria for the scoping review.**

**Inclusion criteria**

- Population: early childhood educators within early childhood education and care (ECEC) centers
- Concept: eHealth (digital) tools
- Context: nutrition and physical activity ECEC environment
- Study type: primary studies with any design or data type
- Study focus: eHealth tool is the primary focus of the resource
- Publication status: peer-reviewed journals and gray literature
- Publication language: English or French
- Full text accessible

**Exclusion criteria**

- Data only collected on children within the ECEC centers
- eHealth technology is a minor component of the assessment or intervention
- Family-based childcare, preschool programs operating less than 4 hours, and before- or after-school care
- Publication language other than English or French
- Full text not available

#### Participants or Population

This review will consider studies that include early childhood educators within ECEC programs, publicly or privately operated, providing full-day care for children aged 0-5 years. Studies will be excluded if educators work in a family-based setting, preschool programs where children attend less than 4 hours per day, or before- or after-school care.

#### Concept

This review will consider studies that explore eHealth tools that aim to support nutrition environments, physical activity environments, or both in the ECEC setting. eHealth tools may be defined using any of the constructs displayed (Textbox 2). eHealth tools include digital technologies that (1) measure and (2) deliver interventions to improve nutrition environments, physical activity environments, or both. Additionally, eHealth tools that assess the ECEC environment must provide feedback to ECECs. We will consider all types of eHealth modalities including but not limited to web-based tools, mobile apps, SMS text messages, social media, and gamification. We will only include studies where the eHealth tool is an essential assessment or intervention component. The eHealth tool should be the predominant mode of assessment or intervention delivery. We will exclude studies in which the eHealth modality constitutes a minor component, for example, if the majority of the assessment or intervention is conducted in person.

**Definitions of the inclusion criteria in the scoping review.**

**Population: early childhood educators within early childhood education and care (ECEC) centers**

- Educators include the staff within an ECEC center with any responsibilities for the care of children aged 0-5 years, which includes teachers or center directors.
- Studies will be excluded if educators work within family-based care, before- and after-school care, or preschools where children attend less than 4 hours per day.

**Concept: eHealth (digital) tools**

- eHealth tools are digital technologies that are noninvasive and used to assess and track the health of a patient or consumer.
- eHealth assessment is used as a term to indicate eHealth tools that measure and evaluate ECECs’ nutrition environment, physical activity environment, or both, policies, knowledge, and practices.
- eHealth intervention is used as a term to indicate eHealth tools that support ECEC centers to improve their nutrition environment, physical activity environment, or both, policies, knowledge, and practices.
- eHealth modalities are eHealth tools such as websites, mobile or smartphone (SMS text messages and apps), emails, digital games, and social media.
- Studies will be excluded if the digital technology is only a minor part of the assessment (ie, web-based survey without feedback to participants) or intervention.

**Context: nutrition and physical activity environment within ECEC centers**

- Focused on the domain of nutrition and physical activity related to child health
- Nutrition environments will be defined in the physical context (eg, availability of healthy food and dramatic play center toys) or the social context (eg, adult-child interactions during meals and ECEC mealtime policies).
- Physical activity environments will be defined in the physical context (eg, space for movement activities and time dedicated to activity) or the social context (eg, adult-child interactions during the outdoor time and ECEC physical activity policies).

#### Context

This review will consider the nutrition and physical activity environments in the ECEC setting as the context. We will focus on both the physical and social environment in ECECs.

#### Information Sources

The information sources for our scoping review include bibliographic databases PubMed, Scopus, CINAHL Plus (EBSCOhost), ERIC (EBSCOhost), and Embase (OVID). These databases were chosen because they host the most relevant literature in public health digital technologies within the education domain. We will also conduct a scan of the gray literature in accordance with Godin et al [39] that includes (1) the gray literature database ProQuest, (2) a customized Google search, and (3) targeted websites to identify unpublished studies and gray literature. This scoping review will consider all relevant literature including quantitative, qualitative, and mixed methods study designs. Systematic reviews, text, and opinion papers will be considered for inclusion in the proposed scoping review if identified.

### Search Strategy

To ensure a comprehensive search, our search strategy will follow the 3-step search strategy from Peters et al [36]. First, an initial limited search of PubMed and Scopus will be undertaken to identify papers on the topic. The text words contained in the titles and abstracts of relevant papers and the index terms used to describe the papers will be used to develop a refined search strategy for PubMed (Multimedia Appendix 1). Second, the search strategy, including all identified keywords and index terms, will be adapted for each included information source. Third, the reference list of retrieved papers will be examined for additional relevant sources. Papers published in English and French with no limit on publication date will be included in this review.

### Study Selection

Following the search, Covidence (Veritas Health Innovation) will be used to upload search results and identify and remove duplicates. A pilot test of 10 papers will be conducted by 2 independent reviewers to assess agreement regarding the relevance of titles and abstracts. Following the pilot test, the reviewers will conduct title and abstract screening against eligibility criteria to determine whether to include the study in the review. In the following step, a full-text screening of selected citations will be done by 2 independent reviewers, and a third reviewer will be consulted in the case of disagreements that cannot be resolved by discussion. Reasons for exclusion of full-text papers that do not meet the inclusion criteria will be recorded and reported in the scoping review. The results of the search will be reported in full in the final scoping review and presented in a PRISMA (Preferred Reporting Items for Systematic Reviews and Meta-Analyses) flow diagram [40].

### Data Extraction

Data will be extracted from papers included in the scoping review by 2 independent reviewers using a data extraction tool developed by the research team. The data extracted will include specific details about the population, concept, context, methods, and key findings relevant to the review question. The research team will use a data extraction tool adapted from the template provided in the Joanna Briggs Institute methodology for scoping reviews [36]. The data extraction form will include paper information (author, publication year, and country), study design and aim, study setting, participant information, inclusion or exclusion criteria, sample size, recruitment method, eHealth modality used, eHealth tool components, psychometric validation, theoretical underpinning, and outcome measures (Multimedia Appendix 2). For the gray literature, the extracted data will include (1) resource details (eg, title of document, author or organization, country, year, resource type, purpose, and participants) and (2) eHealth tool details (eg, type of eHealth tool, tool components, psychometric properties, and nutrition or physical activity measured or improved). The data extraction form will be piloted before implementation to ensure agreement in capturing relevant information. The data extraction form will be revised as necessary as the data extraction proceeds. Modifications will be detailed in the full scoping review. Any disagreements that arise between the reviewers that cannot be solved by discussion or consensus will be resolved by a third reviewer.

### Methodological Quality Appraisal

Methodological quality or risk of bias will not be appraised, which is consistent with guidance on scoping review conduct [36]. This work will inform the next steps on a decision to conduct a systematic review where methodological quality appraisal will be conducted.

## Results

The extracted data will be graphically presented in tables and charts in line with the objective of this scoping review. The presentation of results will be organized by each review question. First, a table of all included studies will be presented providing information on authorship, year of publication, country of origin, study design, aims, participants, and outcomes (Table S1 in Multimedia Appendix 3). A second table will display characteristics of the eHealth tools including eHealth modality used, tool components, and psychometric evidence (Table S2 in Multimedia Appendix 3). Data about the use of theoretical framework will be presented in a third table (Table S3 in Multimedia Appendix 3). A narrative summary will describe the results for each research question and will accompany the tabulated results. Data analysis and presentation will depend on data extraction and thus will be subject to change in case of potential modification of the data extraction form. Any changes will be documented in the review.

This study commenced in May 2023. The scoping review protocol was registered in the Open Science Framework to promote transparency and help avoid unintended duplication of reviews. The search strategy was developed in June 2023 in collaboration with 2 university librarians and was refined in August 2023. The title and abstract screening started in August 2023 and is ongoing. The final results will be submitted to an open-access peer-reviewed journal in 2024.

## Discussion

### Overview

Using eHealth tools can facilitate the evaluation of ECEC environments and the delivery of interventions aimed at enhancing nutrition and physical activity practices within those settings. The synthesis and analysis of existing literature are useful to characterize the current scope of evidence and identify opportunities for future reviews and studies. This protocol will guide a scoping review to identify the extent, range, and characteristics of available evidence [36] on eHealth tools in ECEC settings and to identify any gaps and limitations. This scoping review is also examining the use of theory in the development of the eHealth tools. This information can support the replication of effective tools and inform future intervention development. The results of this scoping review will be disseminated through publication in a peer-reviewed journal.

### Strengths and Limitations

While we aim to conduct a comprehensive scoping review, some limitations should be considered. First, only studies written in English or French will be included. Second, this scoping review is limited to full-time day care centers and excludes home day cares and after-school cares. Family or home day cares are excluded due to the scarcity of formalized information available for this group, and after-school cares are excluded as they do not typically serve children aged 0 to 5 years.

This review has several strengths. First, the search strategy was reviewed by 2 librarians with extensive knowledge in scoping and systematic reviews. Second, our search strategy targets diverse databases that are known to host relevant peer-reviewed literature in public health digital technologies within the education domain. Additionally, the search will be supplemented by gray literature that will broaden the scope of the review. Lastly, this scoping review is the first to synthesize the eHealth tools that assess or improve the nutrition and physical activity environment in early learning settings. The results of this scoping review will provide valuable insights into the potential of eHealth tools to promote healthier environments in early learning settings.

### Conclusions

This scoping review will identify the types of eHealth technologies in the context of health promotion within early learning settings. This knowledge could guide further research on how eHealth technologies can support the promotion of healthy practices in childcare settings and ultimately inform the development of evidence-based interventions to improve health behaviors and outcomes in children.

## Appendix

Multimedia Appendix 1 Search strategy.

Multimedia Appendix 2 Data extraction instrument.

Multimedia Appendix 3 Data presentation tables.

## Abbreviations

ECEC: early childhood education and care
NCD: noncommunicable disease
PRISMA: Preferred Reporting Items for Systematic Reviews and Meta-Analyses
PRISMA-ScR: Preferred Reporting Items for Systematic Reviews and Meta-Analyses Extension for Scoping Reviews
