# Supplementary material for: eHealth Tools Supporting Early Childhood Education and Care Centers to Assess and Enhance Nutrition and Physical Activity Environments: Protocol for a Scoping Review
Source: JMIR Res Protoc. 2023 Oct 24;12:e52252. doi: 10.2196/52252 (PMC10630867; doi:10.2196/52252)
Supplement: Multimedia Appendix 1 [file resprot_v12i1e52252_app1.docx]

PubMed search

Search conducted on September 27, 2023.

| **Search** | **Query** | **Records retrieved** |
| --- | --- | --- |
| #1 | "early childhood education and care"[Title/Abstract] OR "day care*"[Title/Abstract] OR daycare*[Title/Abstract] OR "child care"[Title/Abstract] OR preschool[Title/Abstract] OR "licensed day care"[Title/Abstract] OR "long-term day care"[Title/Abstract] OR nursery[Title/Abstract] OR nurseries[Title/Abstract] OR kindergarten[Title/Abstract] OR "pre-school teacher*"[Title/Abstract] OR ECEC[Title/Abstract] OR educator*[Title/Abstract] OR "Child Care"[Mesh] OR "Child Day Care Centers"[Majr] OR "Child, Preschool"[Majr] OR "Nurseries, Infant"[Mesh] | 126,527 |
| #2 | "e-health"[tw] OR ehealth[tw] OR m-health[tw] OR "online intervention"[tw] OR "online tool" [tw]OR "e-health tool"[tw] OR "digital intervention"[tw] OR "mobile health"[tw] OR "mobile application*"[tw] OR "mobile app"[tw] OR "mobile apps"[tw] OR internet-based[tw] OR "computer based"[tw] OR distance[tw] OR remote[tw] OR "web-based"[tw] OR "online systems"[tw] OR "mobile phone"[tw] OR "smartphone app"[tw] OR "Internet*"[Mesh] OR "Internet-Based Intervention*"[Majr] OR “self-assessment”[Mesh] OR "Mobile Applications*"[Mesh] | 605,229 |
| #3 | "nutrition environment"[Title/Abstract] OR "Menu Planning"[Title/Abstract] OR "Nutrition Policy"[Title/Abstract] OR "Practice guidelines"[Title/Abstract] OR Physical activit*[Title/Abstract] OR “Physical play”[Title/Abstract] OR “active play”[Title/Abstract] OR "Beverages"[Mesh] OR "Nutrition Policy"[Mesh] OR "Diet*"[Mesh] OR "Exercise*"[Mesh] OR “feeding behavior”[Mesh] | 1,821, 227 |
| #4 | #1 AND #2 AND #3 | 369 |
| Limited to English and French | | 361 |
