# Supplementary material for: eHealth Tools Supporting Early Childhood Education and Care Centers to Assess and Enhance Nutrition and Physical Activity Environments: Protocol for a Scoping Review
Source: JMIR Res Protoc. 2023 Oct 24;12:e52252. doi: 10.2196/52252 (PMC10630867; doi:10.2196/52252)
Supplement: Multimedia Appendix 2 [file resprot_v12i1e52252_app2.docx]

| Study ID (author, date) |  |
| --- | --- |
| Study title |  |
| Country |  |
| Study aim |  |
| Study design |  |
| Study setting |  |
| Participant information (age/sex) |  |
| Inclusion/exclusion criteria |  |
| Sample size |  |
| Method of recruitment |  |
| Type of eHealth tool (web-based, app-based, SMS) |  |
| Tool components (what does it assess or support) |  |
| Details of psychometric validation of tool |  |
| Theoretical underpinning |  |
| Outcome measures |  |
