# Supplementary material for: eHealth Tools Supporting Early Childhood Education and Care Centers to Assess and Enhance Nutrition and Physical Activity Environments: Protocol for a Scoping Review
Source: JMIR Res Protoc. 2023 Oct 24;12:e52252. doi: 10.2196/52252 (PMC10630867; doi:10.2196/52252)
Supplement: Multimedia Appendix 3 [file resprot_v12i1e52252_app3.docx]

**Table S1.** Characteristics of included studies.

| Author and year of publication | Country | Study design | Study aim | Study setting | Sample size (at baseline) | Gender of overall participants (N % of Females) | Age of participants | Outcome measures |
| --- | --- | --- | --- | --- | --- | --- | --- | --- |
|  |  |  |  |  |  |  |  |  |

**Table S2.** Characteristics of eHealth tools.

| Study | eHealth modality (web, application, SMS etc.) | Assessment components | Psychometric evidence | Intervention components | Length of intervention |
| --- | --- | --- | --- | --- | --- |
|  |  |  |  |  |  |

**Table S3.** Theoretical framework.

| Study | Theory-based eHealth tool (Y/N) | Theory used |
| --- | --- | --- |
|  |  |  |
